# Supplementary material for: Pharmacologically controlling protein-protein interactions through epichaperomes for therapeutic vulnerability in cancer
Source: Commun Biol. 2021 Nov 25;4:1333. doi: 10.1038/s42003-021-02842-3 (PMC8617294; doi:10.1038/s42003-021-02842-3)
Supplement: Supplementary file 2 — Supplementary Information [file 42003_2021_2842_MOESM2_ESM.pdf]

Supplementary Information

Pharmacologically controlling protein-protein interactions through epichaperomes for therapeutic vulnerability in cancer

Joshi et al.

Contains

Supplementary Figures 1 through 18

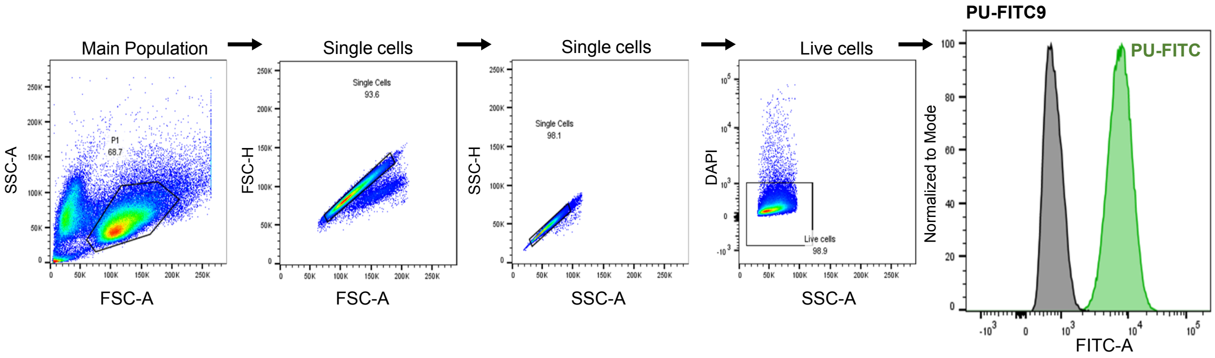

Supplementary Fig. 1: Gating strategy for epichaperome determination using PU-FITC flow cytometry. PU-FITC, epichaperome probe; FITC9, negative control. Epichaperome detection in MDA-MB-468 cancer cells is shown as an example.

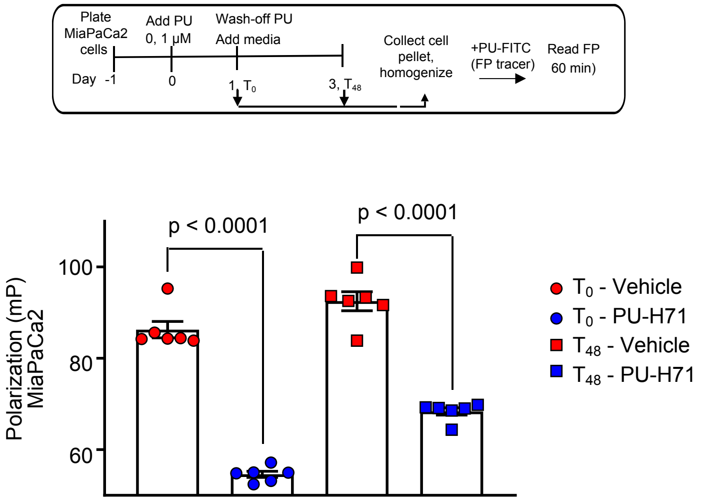

Supplementary Fig. 2: Baseline active epichaperomes remain occupied by PU-H71 even after its removal from the cell culture media.

Fluorescence polarization (FP) analysis performed in MiaPaCa2 cell lysates pre-incubated with PU-H71 (1 µM) or vehicle as indicated in the schematic. n = 6 biological replicates from 3 independent experiments. Graph, mean ± SEM; unpaired two-tailed t-tests, V vs PU at time 0 h and time 48 h.

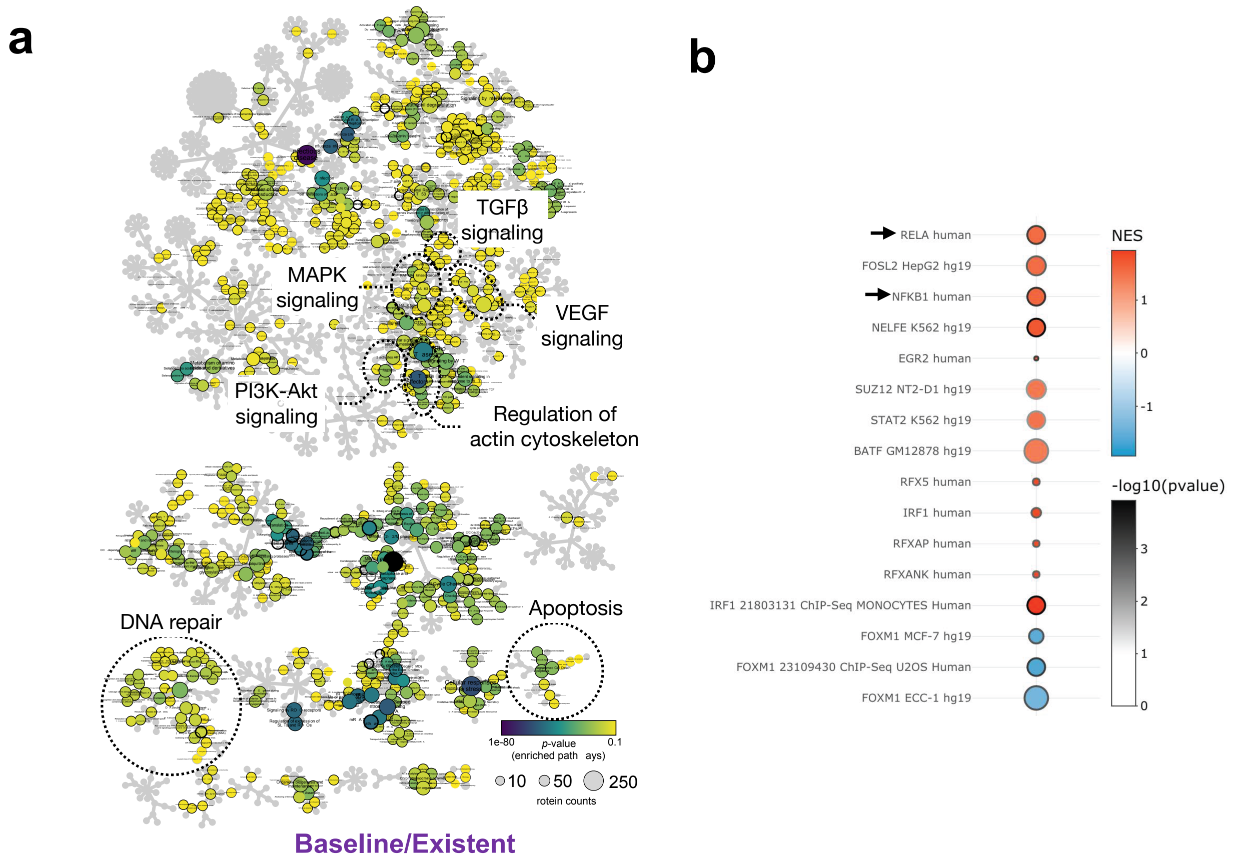

Supplementary Fig. 3: Omics analyses of the baseline and deployed cellular states.
a Functional mapping of MiaPaCa2 at baseline through epichaperomics. Systems level analysis of protein pathways regulated by epichaperomes at baseline (MiaPaCa2) as determined by epichaperomics. Reactome pathways analysis is shown. **b** RNA-seq data analysis of baseline and deployed states. Gene Set Enrichment Analysis (GSEA) performed on MiaPaCa2-PU versus MiaPaCa2 datasets (see Fig. 4, baseline/existent vs deployed) using transcriptional factor related pathway databases. Enrichr enrichment of upregulated and downregulated genes (see methods).The gene set was pre-ranked by logFC prior to the test. The dot size is propositional to setSize (GSEA) and the filled color represents the Normalized Enrichment Score (NES) value. The border color gradient represents the -log10 transformed p.adj of GSEA. Only enriched TFs (adj.P<0.1) are shown.

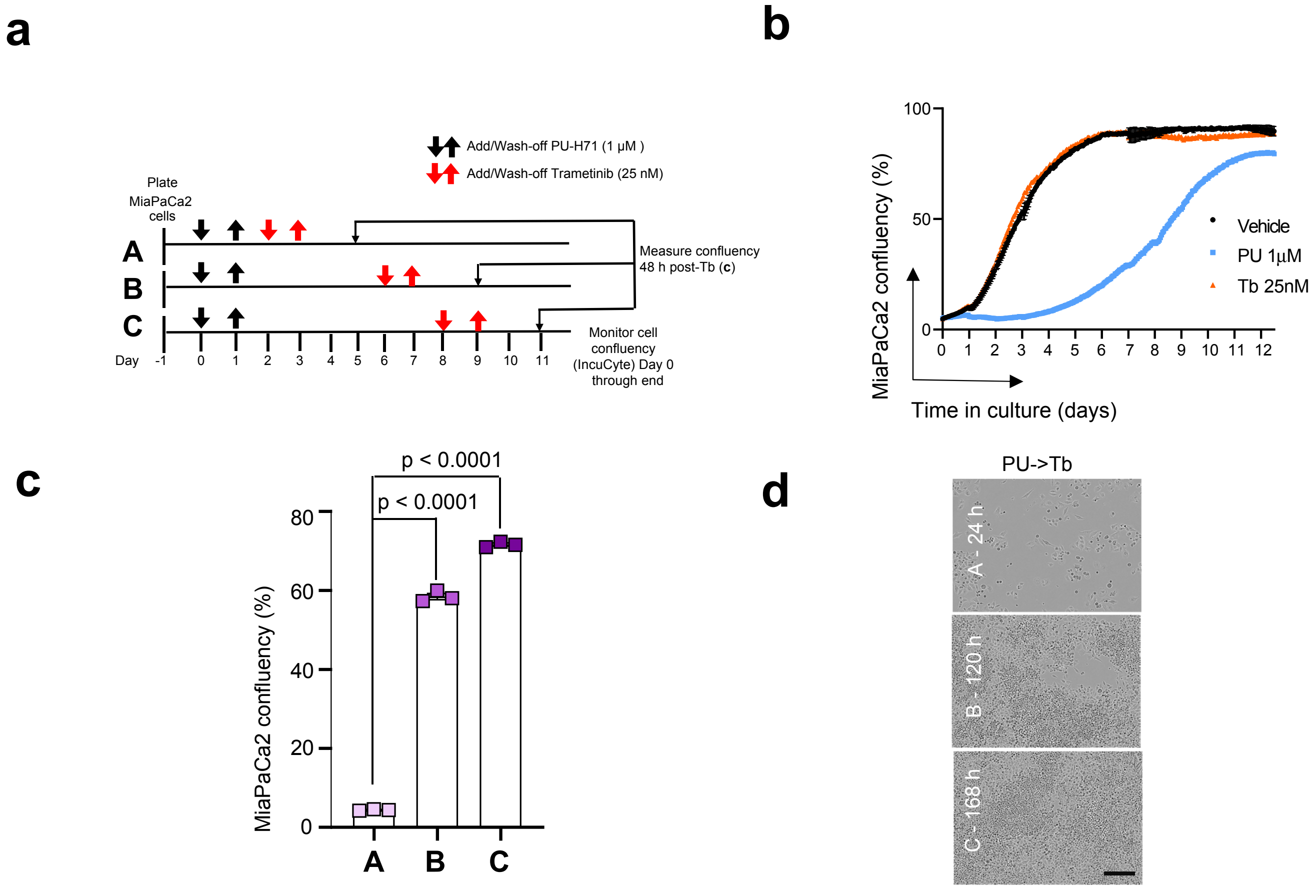

Supplementary Fig. 4: Addition of Trametinib after the engineered hyperconnectivity state is lost, is less effective than if Trametinib is added when cells are in the hyperconnectivity state.
a Schematic showing the experimental design. A, B and C: Trametinib (25 nM) added at 24 h (i.e. hyperconnectivity state), 120 h or 168 h (i.e. in recovery from and post-hyperconnectivity state), respectively, post PU-H71 (1 µM) wash-off. **b** Cell confluency and viability as per (**a**) monitored by live cell microscopy for single agent addition. **c** As in (**a**,**b**) sequential PU->Tb treatment with confluency measured at 48 h post Trametinib wash-off. Error bars mean ± SEM; n = 3 biological replicates, one-way ANOVA with Dunnett's post-hoc. **d** Micrographs in are representative of each experimental condition from (**c**). Scale bar, 300 µm.

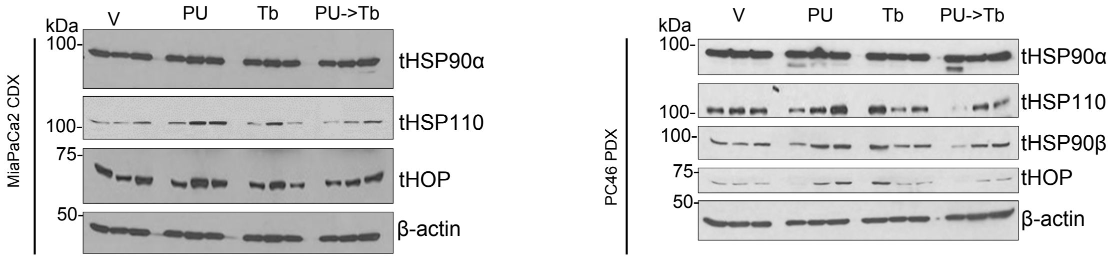

Supplementary Fig. 5: Chaperone levels.

Total levels of chaperome components in the indicated experimental conditions as in Fig. 8 (n = 3 per mouse cohort). Each column represents and individual tumour.

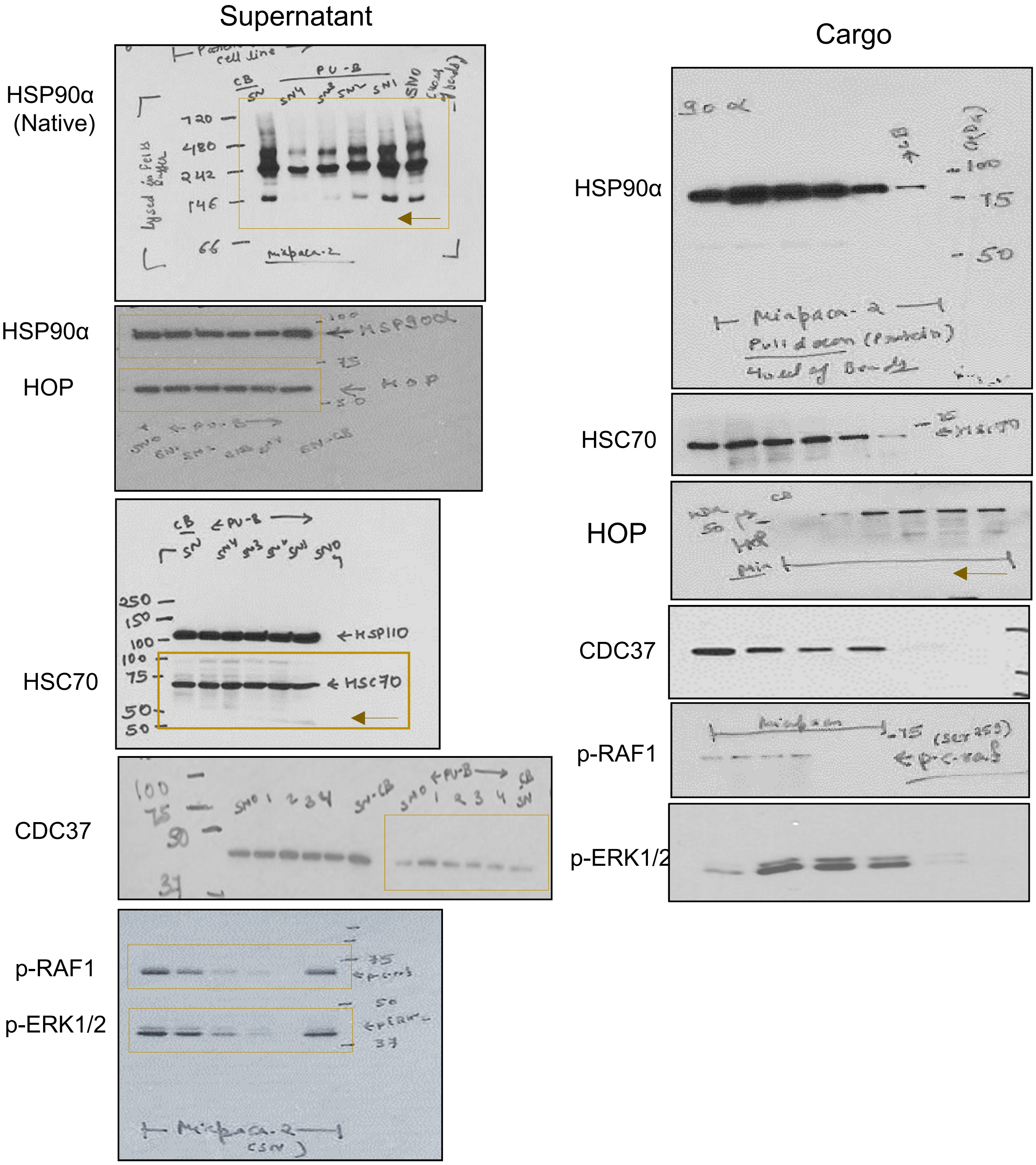

Supplementary Fig. 6: Uncropped images.
Membranes used for immunodetection shown in Fig. 1g.

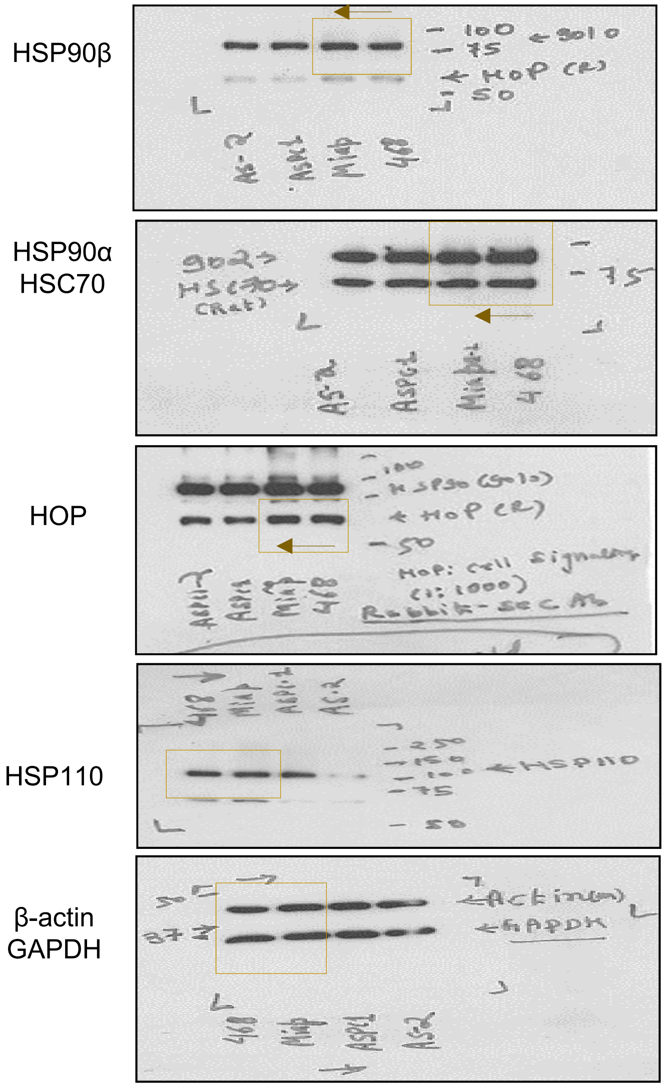

Supplementary Fig. 7: Uncropped images.
Membranes used for immunodetection shown in Fig. 1i.

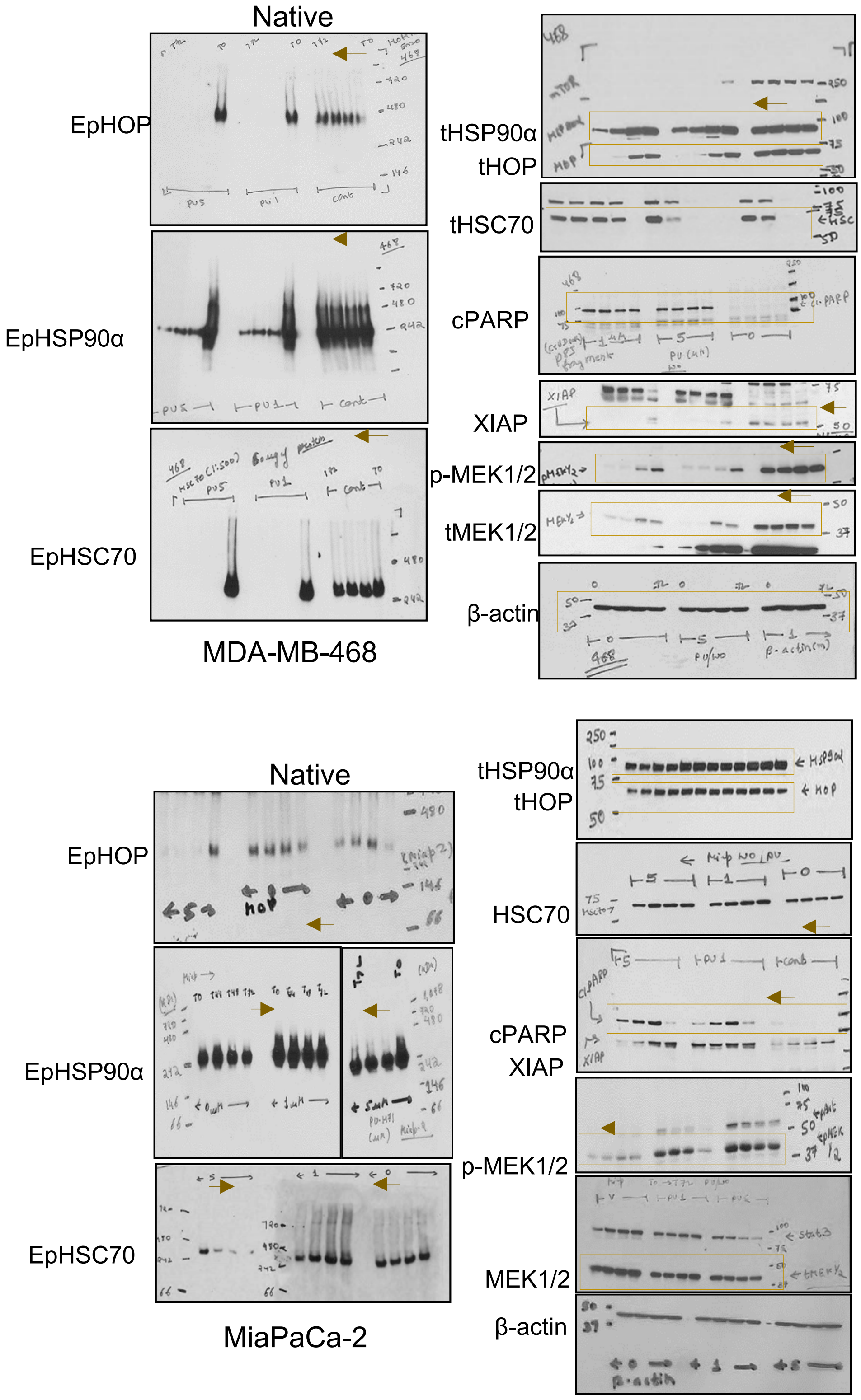

Supplementary Fig. 8: Uncropped images.
Membranes used for immunodetection shown in Fig. 2a.

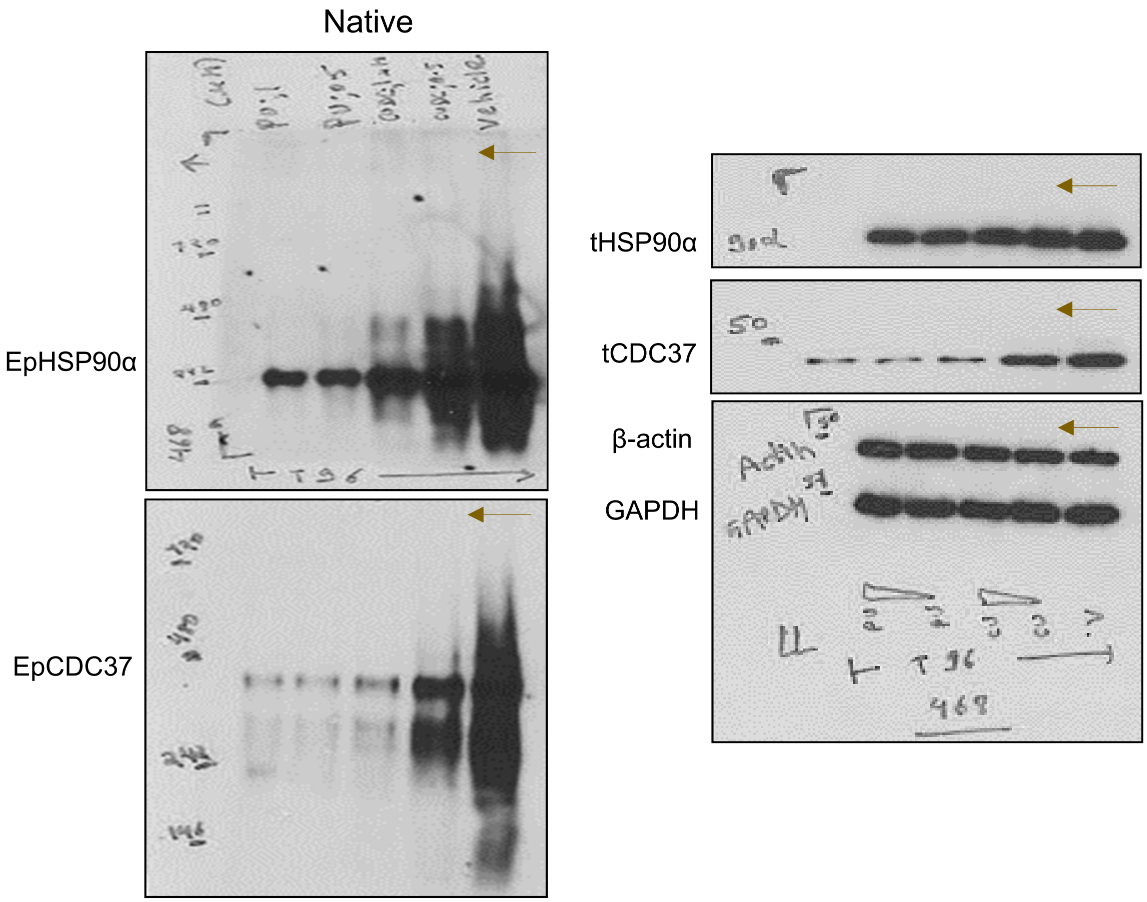

Supplementary Fig. 9: Uncropped images.
Membranes used for immunodetection shown in Fig. 2e.

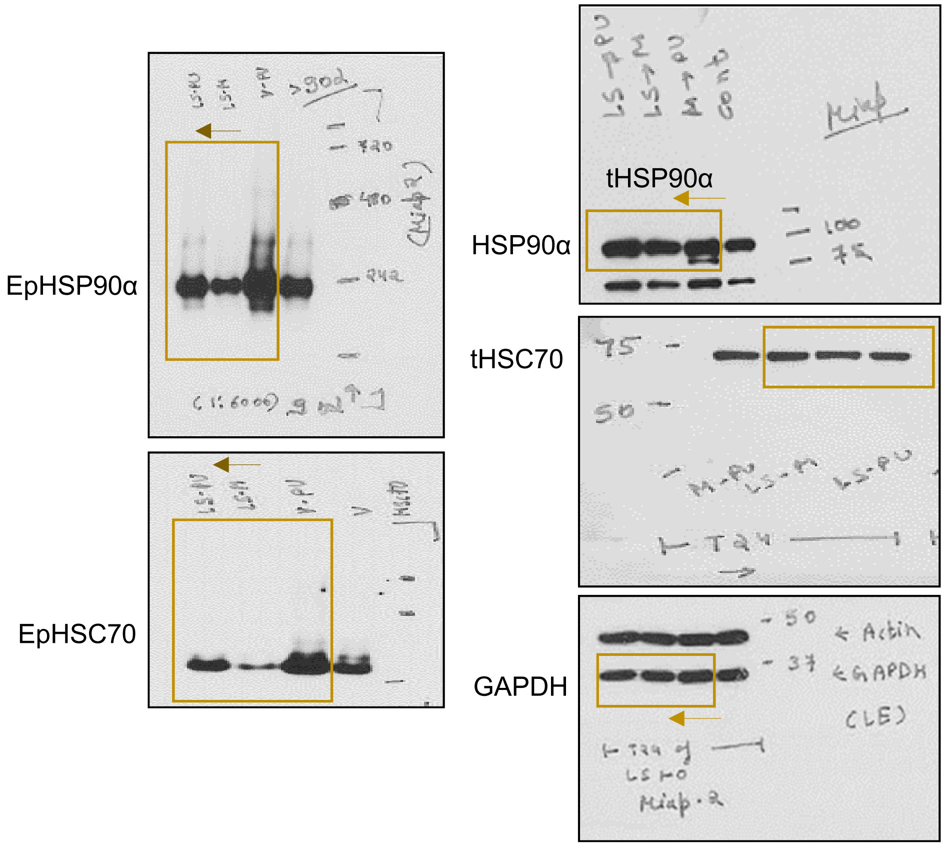

Supplementary Fig. 10: Uncropped images.
Membranes used for immunodetection shown in Fig. 3d.

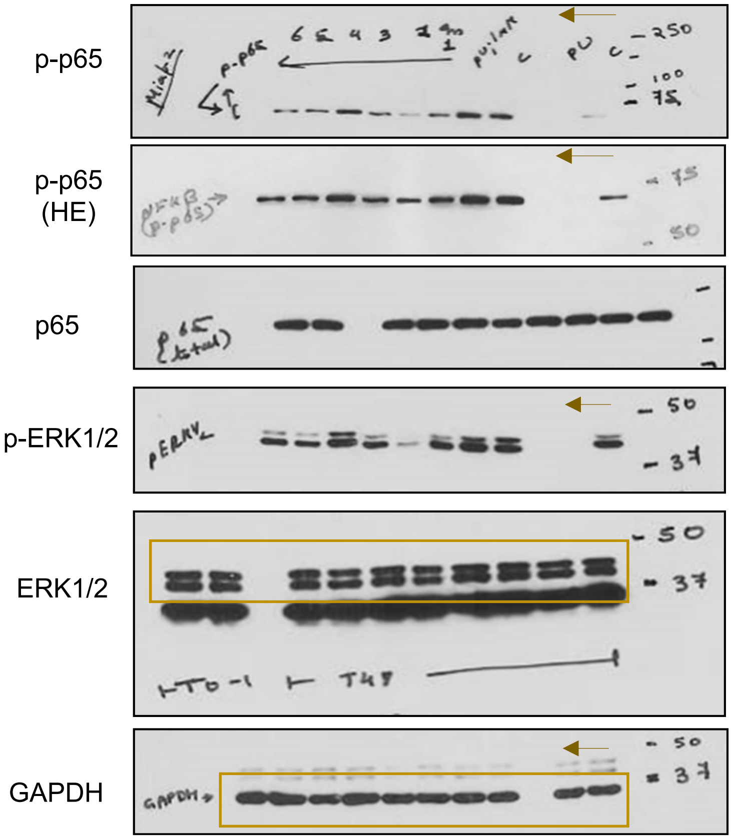

Supplementary Fig. 11: Uncropped images.
Membranes used for immunodetection shown in Fig. 4c.

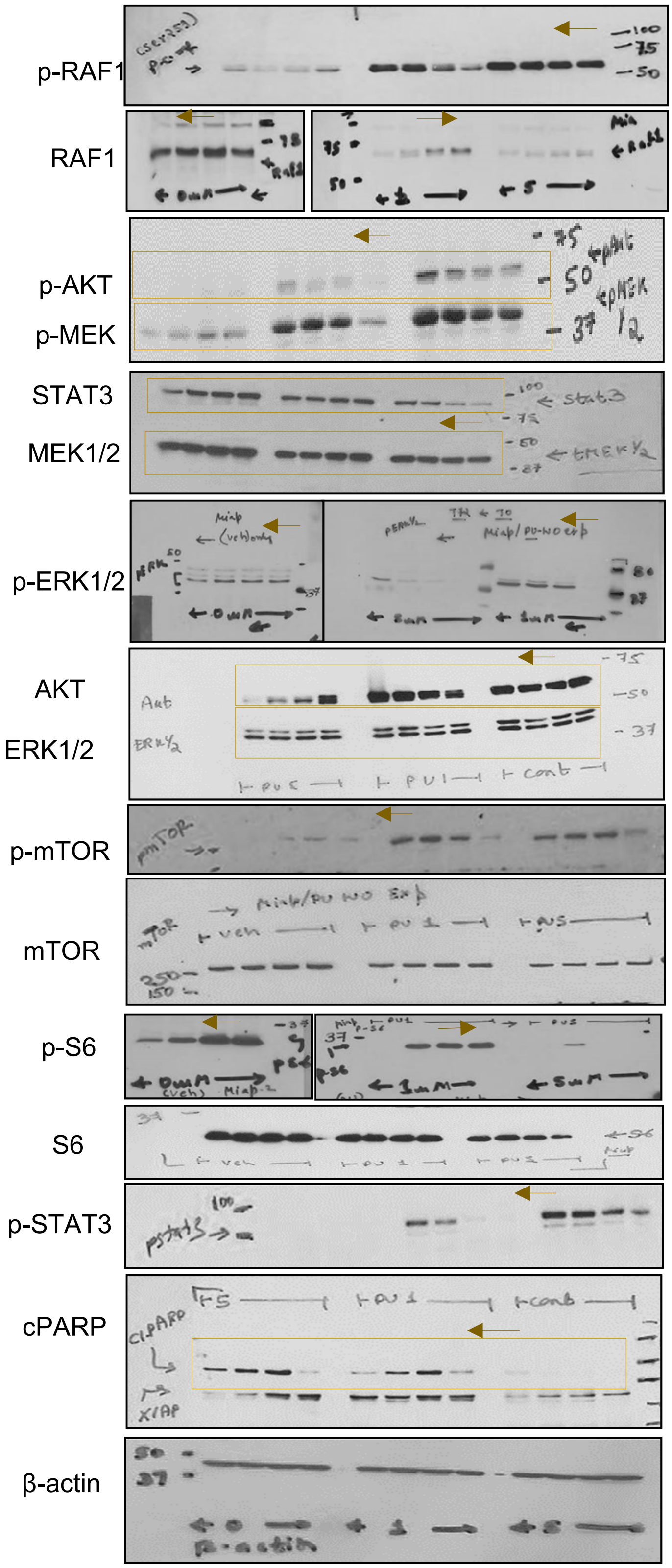

Supplementary Fig. 12: Uncropped images.
Membranes used for immunodetection shown in Fig. 5a.

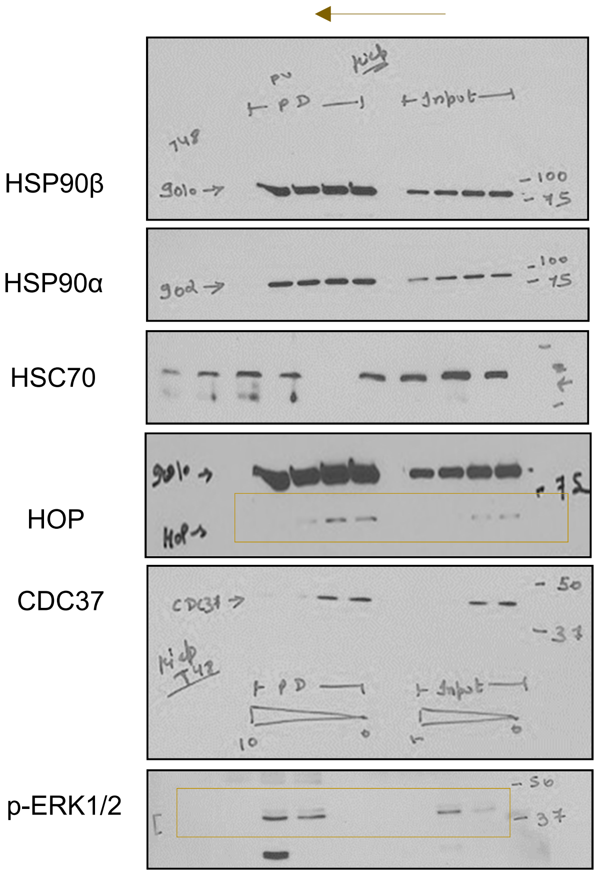

Supplementary Fig. 13: Uncropped images.
Membranes used for immunodetection shown in Fig. 5b.

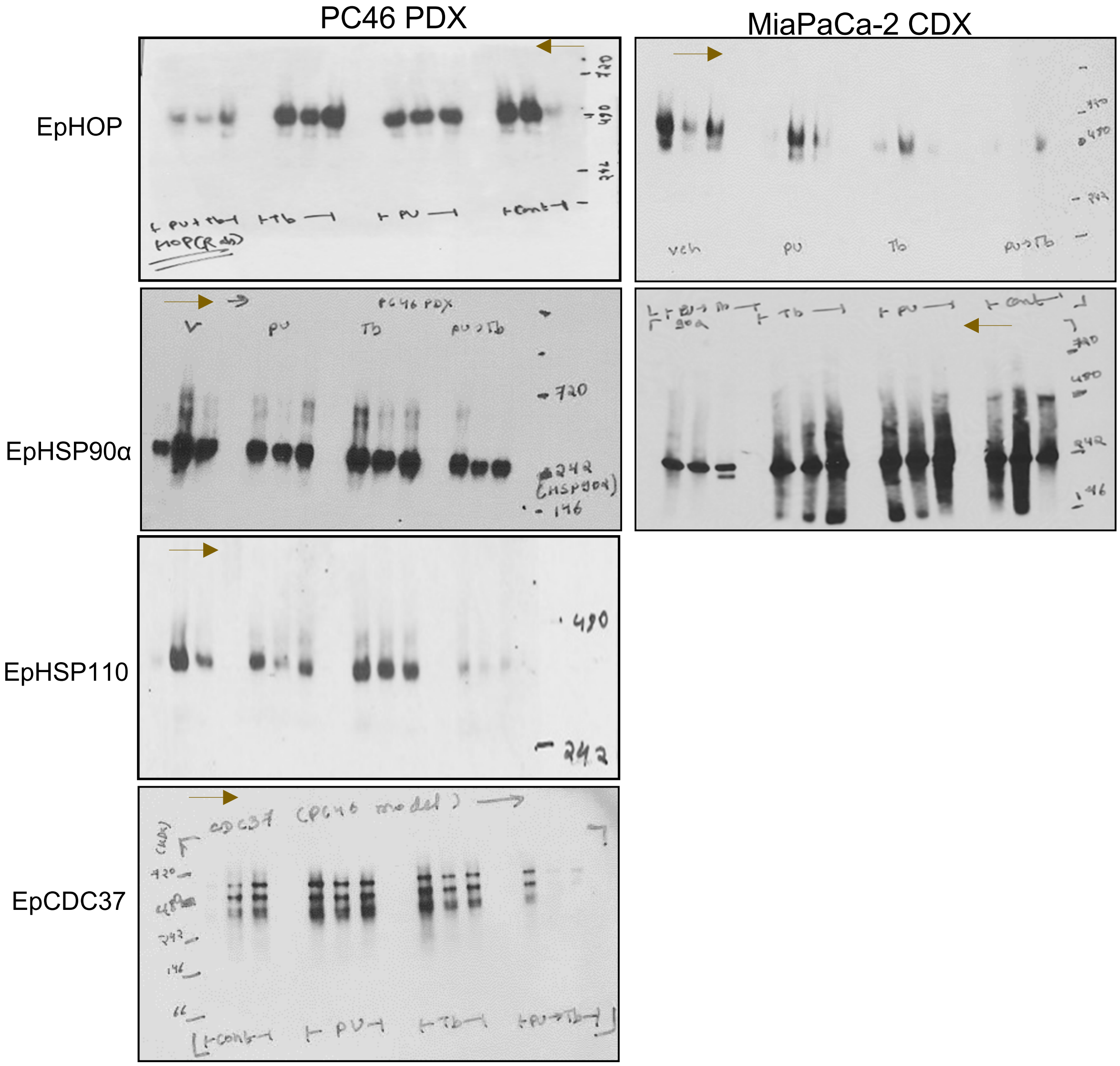

Supplementary Fig. 14: Uncropped images.
Membranes used for immunodetection shown in Fig. 8b.

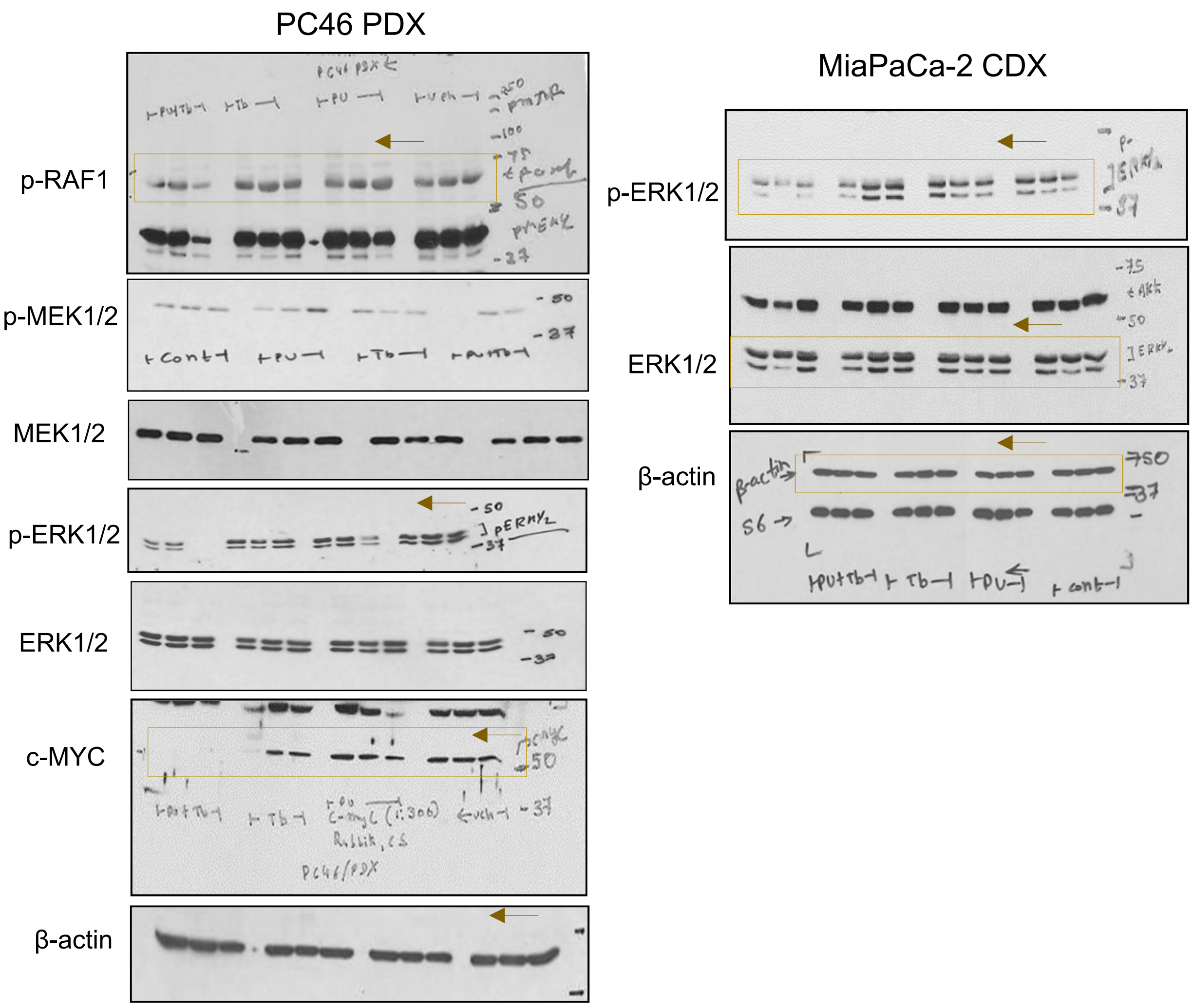

Supplementary Fig. 15: Uncropped images.
Membranes used for immunodetection shown in Fig. 8b.

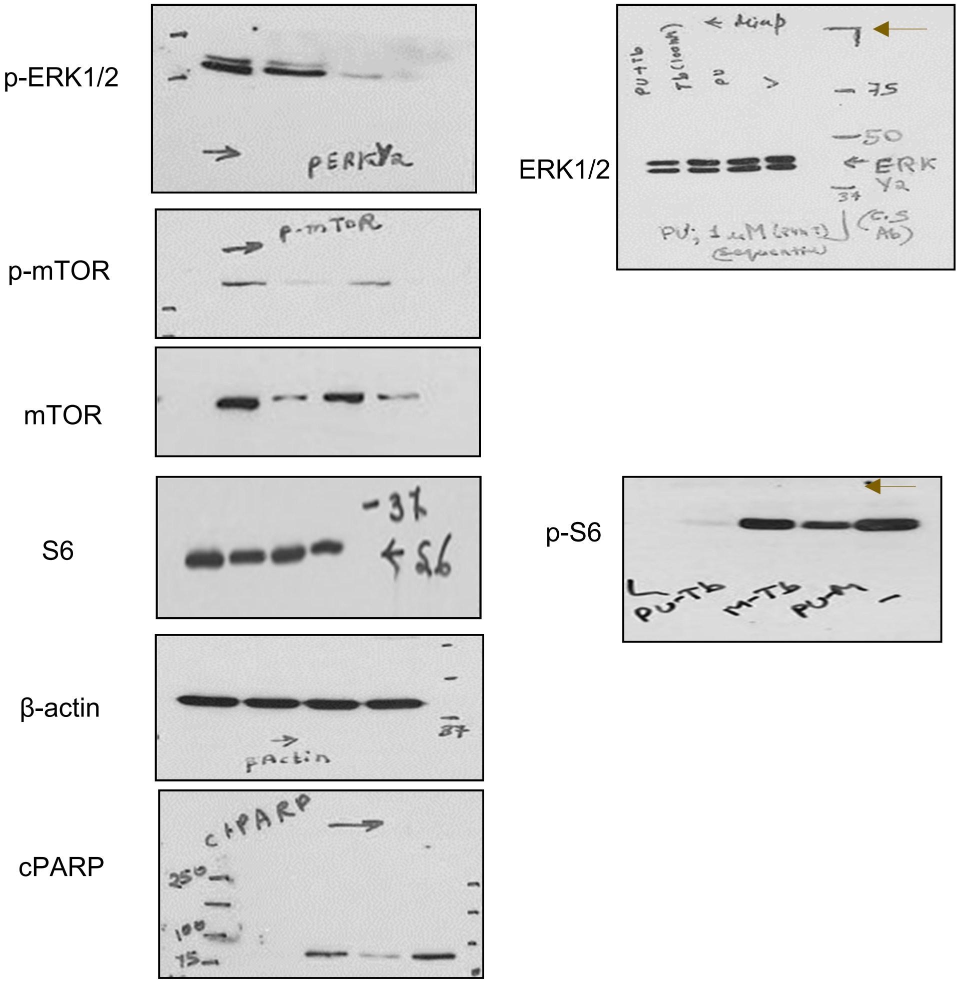

Supplementary Fig. 16: Uncropped images.
Membranes used for immunodetection shown in Fig. 9a.

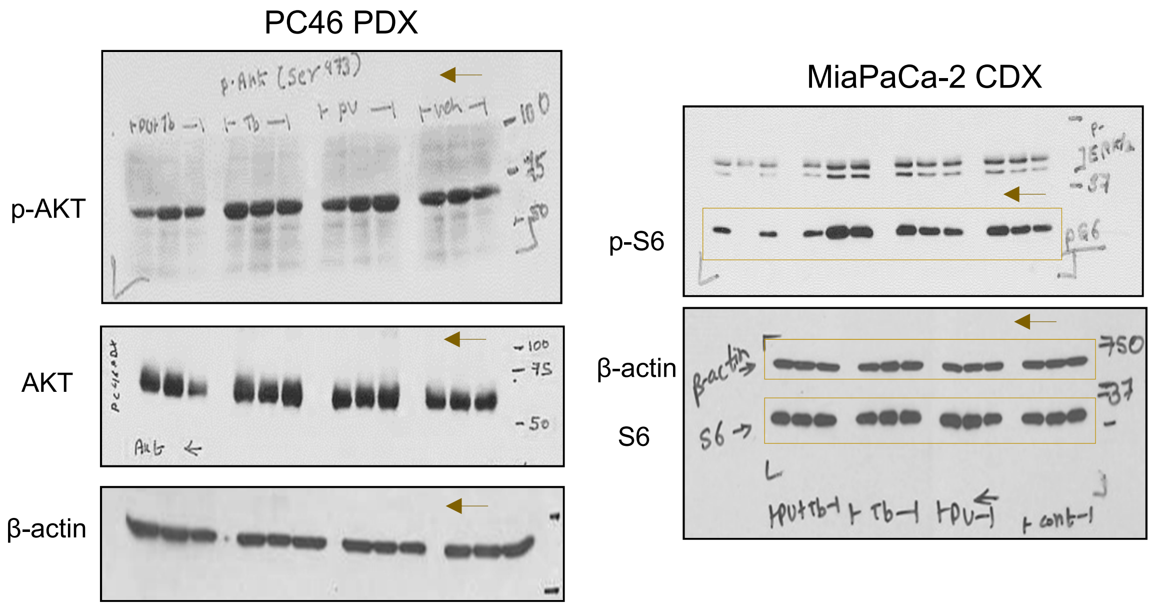

Supplementary Fig. 17: Uncropped images.
Membranes used for immunodetection shown in Fig. 9b.

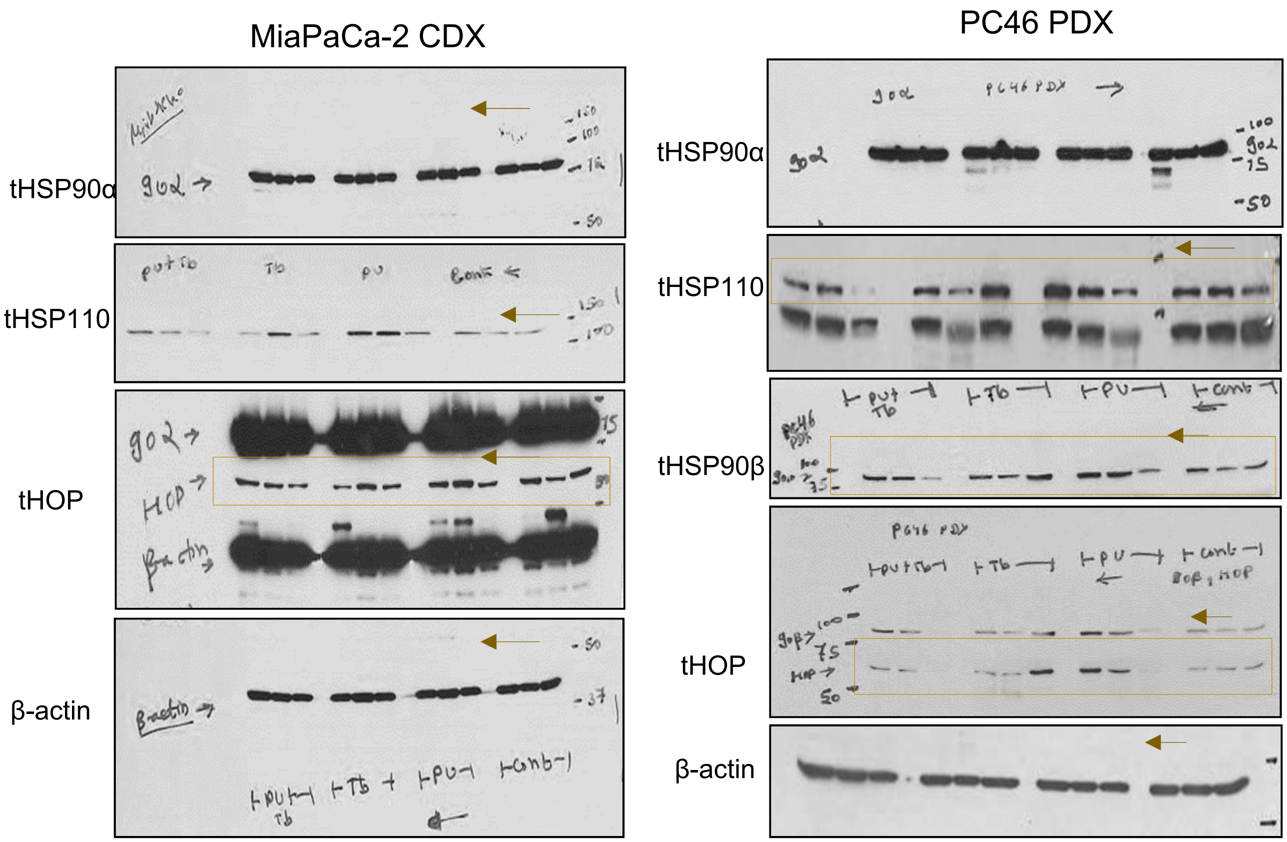

Supplementary Fig. 18: Uncropped images.
Membranes used for immunodetection shown in Supplementary Fig. 5.
