# Supplementary material for: Pharmacologically controlling protein-protein interactions through epichaperomes for therapeutic vulnerability in cancer
Source: Commun Biol. 2021 Nov 25;4:1333. doi: 10.1038/s42003-021-02842-3 (PMC8617294; doi:10.1038/s42003-021-02842-3)
Supplement: Supplementary file 3 — Description of Additional Supplementary Files [file 42003_2021_2842_MOESM3_ESM.pdf]

Description of Additional Supplementary Files

File name: Supplementary Data 1
Description: Epichaperomics dataset analyses as per Fig. 3a.

File name: Supplementary Data 2
Description: RNAseq dataset analyses as per Supplementary Fig. 3b.

File name: Supplementary Data 3
Description: All source data underlying the graphs and charts presented in the figures.
