# Supplementary material for: Pharmacologically controlling protein-protein interactions through epichaperomes for therapeutic vulnerability in cancer
Source: Commun Biol. 2021 Nov 25;4:1333. doi: 10.1038/s42003-021-02842-3 (PMC8617294; doi:10.1038/s42003-021-02842-3)
Supplement: Supplementary file 7 — Reporting Summary [file 42003_2021_2842_MOESM7_ESM.pdf]

nature research

Corresponding author(s): Gabriela Chiosis

Last updated by author(s): Oct 18, 2021

Reporting Summary

Nature Research wishes to improve the reproducibility of the work that we publish. This form provides structure for consistency and transparency in reporting. For further information on Nature Research policies, see Authors & Referees and the Editorial Policy Checklist .

Statistics

For all statistical analyses, confirm that the following items are present in the figure legend, table legend, main text, or Methods section.

n/a	Confirmed	
☐	☒	The exact sample size (*n*) for each experimental group/condition, given as a discrete number and unit of measurement
☐	☒	A statement on whether measurements were taken from distinct samples or whether the same sample was measured repeatedly
☐	☒	The statistical test(s) used AND whether they are one- or two-sided<br>*Only common tests should be described solely by name; describe more complex techniques in the Methods section.*
☒	☐	A description of all covariates tested
☐	☒	A description of any assumptions or corrections, such as tests of normality and adjustment for multiple comparisons
☐	☒	A full description of the statistical parameters including central tendency (e.g. means) or other basic estimates (e.g. regression coefficient) AND variation (e.g. standard deviation) or associated estimates of uncertainty (e.g. confidence intervals)
☐	☒	For null hypothesis testing, the test statistic (e.g. *F*, *t*, *r*) with confidence intervals, effect sizes, degrees of freedom and *P* value noted<br>*Give P values as exact values whenever suitable.*
☒	☐	For Bayesian analysis, information on the choice of priors and Markov chain Monte Carlo settings
☒	☐	For hierarchical and complex designs, identification of the appropriate level for tests and full reporting of outcomes
☐	☒	Estimates of effect sizes (e.g. Cohen's *d*, Pearson's *r*), indicating how they were calculated

Our web collection on statistics for biologists contains articles on many of the points above.

Software and code

Policy information about availability of computer code

Data collection	FP measurements were performed on an Analyst GT instrument (Molecular Devices, Sunnyvale, CA). For western blotting, the chemiluminescent signal was visualized with Enhanced Chemiluminescence System (GE Healthcare) following manufacturer’s instructions and quantified using image Studio Lite Ver. 5.2 (LI-COR Biosciences). The mean fluorescence intensity (MFI) was measured by flow cytometry on LSRFortessa (BD Biosciences). The FITC derivative FITC9 was used as a negative control. The acquired data was saved into a flow cytometry standard file and analysed using Flow Jo software (FlowJo LLC). The IncuCyte live cell microscopy system (Essen BioScience) was used to evaluate cell confluency. LC-MS/MS analysis was performed using a Q Exactive mass spectrometer coupled to a Thermo Scientific EASY-nLC 1000 (Thermo Fisher Scientific, Waltham, MA) equipped with a self-packed 75 µm x 20-cm reverse phase column (ReproSil-Pur C18, 3 µm, Dr. Maisch GmbH, Germany) for peptide separation. RNA samples were quantified using Qubit 2.0 Fluorometer (Life Technologies, Carlsbad, CA, USA) and RNA integrity was checked using Agilent TapeStation 4200 (Agilent Technologies, Palo Alto, CA, USA).
Data analysis	Prism was used for statistical testings (t-tests and ANOVA). ImageJ (versions 1.4 and 1.52) was used for western blot quantification. SoftMaxPro6 was used for fluorescence polarization data analyses. For flow cytometry data were analysed using Flow Jo software (FlowJo LLC). SynergyFinder 2.0 (https://synergyfinder.fimm.fi) was used to calculate the degree of combination synergy. Cell confluency was measured using the IncuCyte software, where values were pooled, and the mean used to plot each datapoint on the graph. Data were imported and analysed in GraphPad Prism 8. For RNAseq, the R package DESeq2 was used to determine the significant differentially expressed genes and calculate the log2 fold change of normalized mean hit counts in MiaPaCa-PU versus MiaPaCa. Gene annotations were downloaded from hsapiens_gene_ensembl database (version GRCh38.p13) via R package biomaRt (version 2.48.3) and all ensemble gene ids were mapped to hgnc symbol IDs for further analysis. For those hgnc symbols with multiple corresponding ensemble IDs, we aggregated them and took the mean value. Then, a universal gene set enrichment analysis was conducted via R package clusterProfiler (GSEA function; version 4.0.5). During the analysis, the DE gene set pre-ranked by their log2 fold change values were compared against three annotated transcript factor gene sets downloaded from Enrichr libraries (https://maayanlab.cloud/Enrichr/; TRRUST_Transcription_Factors_2019_enricher, ChEA_2016_enricher, ENCODE_TF_ChIP-seq_2015). For MS data processing, all mass spectra were first converted to mgf peak list format using Proteome Discoverer 1.4 and the resulting mgf files searched against a human UniProt protein database using Mascot (Matrix Science, London, UK; version 2.5.0; www.matrixscience.com). Decoy protein sequences

with reversed sequence were added to the database to allow for the calculation of false discovery rates (FDR). MudPit scoring was typically applied using significance threshold score p<0.01. Decoy database search was always activated and, in general, for merged LS-MS/MS analysis of a gel lane with p<0.01, FDR averaged around 1%. Mascot search results were imported into Scaffold (Proteome Software, Inc., Portland, OR; version 4.7.3) to further analyse tandem mass spectrometry (MS/MS) based protein and peptide identifications. X! Tandem (The GPM, thegpm.org; version CYCLONE (2010.12.01.1) was then performed and its results were merged with those from Mascot. The two search engine results were combined and displayed at 1% FDR. Mass spectra files were also analysed using the MaxQuant proteomics data analysis workflow (version 1.6.0.1) with the Andromeda search engine. Raw mass spectrometer files were used to extract peak lists which were searched with the Andromeda search engine against human or mouse proteome and a file containing contaminants such as human keratins. LFQ intensity (from Maxquant) was used for protein quantitation. To prepare the PPI database, we combined all entries from BioGrid (v.3.4.160) and IntAct (version 05.2018) to create a dataset that inventories all documented human-human (Homo sapiens) PPIs. The interactome network was built in Cytoscape v3.61. For RNAseq, image analysis and base calling were conducted by the HiSeq Control Software (HCS). Raw sequence data (.bcl files) generated from Illumina HiSeq was converted into fastq files and de-multiplexed using Illumina's bcl2fastq 2.17 software. One mismatch was allowed for index sequence identification. After investigating the quality of the raw data, sequence reads were trimmed to remove possible adapter sequences and nucleotides with poor quality using Trimmomatic v.0.36. The trimmed reads were mapped to the Homo sapiens reference genome available on ENSEMBL using the STAR aligner v.2.5.2b. Data analysis, statistical testing and visualization were conducted in R (version 3.5.1; R Foundation for Statistical Computing) or Prism (version 8 or 9). The code for iGSEA is available in Github (github.com/chiosislab/Chaperomics_controllability_2020) and zenodo (https://doi.org/10.5281/zenodo.5585352). It is also explained and cited in the appropriate methods section and ref. 22.

For manuscripts utilizing custom algorithms or software that are central to the research but not yet described in published literature, software must be made available to editors/reviewers. We strongly encourage code deposition in a community repository (e.g. GitHub). See the Nature Research guidelines for submitting code & software for further information.

Data

Policy information about availability of data

All manuscripts must include a data availability statement. This statement should provide the following information, where applicable:

- Accession codes, unique identifiers, or web links for publicly available datasets
- A list of figures that have associated raw data
- A description of any restrictions on data availability

Epichaperomics LC-MS data that support the findings of this study have been deposited in MassIVE with the MSV000085630 accession number. Epichaperomics-derived interactomes are provided in Supplementary Data 1. Epichaperomics related files (dependent libraries, chaperomics proteinGroups input, PPI networks, pathway databases and epichaperomics datasets), final data outputs and visualizations (by Cytoscape) and other Cytoscape files are available in GitHub (github.com/chiosislab/Chaperomics_controllability_2020) and zenodo (https://doi.org/10.5281/zenodo.5585352). The results of pathway enrichment analyses were also included into the Cytoscape files (https://doi.org/10.5281/zenodo.5585352). RNAseq dataset have been deposited in GSA-Human with the HRA001454 accession number. Associated analyses are provided in Supplementary Data 2. The source data underlying the figures can be accessed in Supplementary Data 3. Uncropped and unedited images of the membranes used for immunodetection are available in Supplementary Fig. 6-18. Reagents are available under an MTA with Memorial Sloan Kettering; address requests to G.C.

Field-specific reporting

Please select the one below that is the best fit for your research. If you are not sure, read the appropriate sections before making your selection.

☒ Life sciences ☐ Behavioural & social sciences ☐ Ecological, evolutionary & environmental sciences

For a reference copy of the document with all sections, see nature.com/documents/nr-reporting-summary-flat.pdf

Life sciences study design

All studies must disclose on these points even when the disclosure is negative.

Sample size	No statistical methods were used to predetermine sample size for in vitro but these are similar to those generally employed in the field. For mouse studies, sample sizes were determined by magnitude and consistency of measurable differences in a pilot study we conducted. Sample sizes for in vitro and in vivo experiments are fully disclosed in the manuscript. Experiments were performed in at least 3 biological replicates to sufficiently detect statistical significance, and the number of technical replicates for in vitro studies were determined according to manufacturers' instructions for data acquisitions.
Data exclusions	No data were excluded from the analyses.
Replication	All in vitro and in vivo experiments were performed, in at least 3 biological replicates, with biological and technical replicates which are fully disclosed in the manuscript. Several alternative methods were used to validate observations. Experiments were also replicated through multiple cohort analyses. Results shown are representative of several independently performed experiments (see figure legends, at least 3). There were no findings that could not be replicated or reproduced.
Randomization	Mice were randomized prior to treatment with vehicle control or compounds. For in vitro experiments, samples were allocated into experimental groups by randomization.
Blinding	Investigators were not blinded to group allocation during data collection and/or analysis for the preclinical studies. All values were determined by methods that are independent of operator bias. Mice treatments were not blinded since most of the experiments required daily treatments and treatment groups and mice cage numbers had to be known for investigators. Blinding was not relevant to the remaining

experiments described in the study due to primary investigators performing experiments from start to finish due to technicality required.

Reporting for specific materials, systems and methods

We require information from authors about some types of materials, experimental systems and methods used in many studies. Here, indicate whether each material, system or method listed is relevant to your study. If you are not sure if a list item applies to your research, read the appropriate section before selecting a response.

Materials & experimental systems

n/a	Involved in the study
☐	☒ Antibodies
☐	☒ Eukaryotic cell lines
☒	☐ Palaeontology
☐	☒ Animals and other organisms
☒	☐ Human research participants
☒	☐ Clinical data

Methods

n/a	Involved in the study
☒	☐ ChIP-seq
☐	☒ Flow cytometry
☒	☐ MRI-based neuroimaging

Antibodies

Antibodies used

All antibodies and relevant information is provided in the Methods. Antibodies are listed below with target, clone, supplier, dilution and catalog number: HSP90α (ab2928; RRID:AB_303423; 1:6,000), HSP70 (ab94368; RRID:AB_10716913; 1:5,000) and XIAP (ab21278; RRID:AB_446157; 1:1,000) from Abcam; HSP90β (SMC-107; RRID:AB_854214; 1:2,000) and HSP110 (SPC-195; RRID:AB_2119373; 1:1,000) from Stressmarq; HSP70 (SPA-810; RRID:AB_10616513; 1:1,000), HSC70 (SPA-815; RRID:AB_10617277; 1:1,000), and HOP (SRA-1500; RRID:AB_10618972; 1:1,000) from Enzo; cleaved PARP (or PARP p85 Fragment pAb) (G7341; RRID:AB_430876; 1:1,000) from Promega; p-AKT (S473) (9271; RRID:AB_329825; 1:1,000), AKT (9272; RRID:AB_329827; 1:2,000), p-RAF1 (S259) (9421; RRID:AB_330759; 1:1,000), RAF1 (12552; RRID:AB_2728706; 1:500), p-MEK1/2 (S217/221) (9154; RRID:AB_2138017; 1:1,000), MEK1/2 (9122; RRID:AB_823567; 1:1,000), p-ERK1/2 (T202/Y204) (4377; RRID:AB_331775; 1:1,000), ERK1/2 (4695; RRID:AB_390779; 1:2,000), p-STAT3 (Y705) (9145; RRID:AB_2491009; 1:1,000), STAT3 (9139; RRID:AB_331757; 1:2,000), p-p65 (Ser536) (3033; RRID:AB_331284; 1:500), p65 (8242; RRID:AB_10859369; 1:2,000), p-mTOR (S2448) (5536; RRID:AB_10691552; 1:500), mTOR (2983; RRID:AB_2105622; 1:1,000), CDC37 (4793; RRID:AB_10695539; 1:1,000), CHIP (2080; RRID:AB_2198052; 1:2,000), HOP (5670; RRID:AB_10828378; 1:1,000), p-S6 ribosomal protein (Ser235/236) (4858; RRID:AB_916156; 1:2,000), S6 ribosomal protein (2217; RRID:AB_331355; 1:3,000) and GAPDH (2118; RRID:AB_561053; 1:4,000) from Cell Signaling Technology and β-actin (A1978, RRID:AB_476692; 1:6,000) from Sigma-Aldrich, HSP90β (SMC-107; RRID:AB_854214; 1:2,000) and HSP110 (SPC-195; RRID:AB_2119373; 1:1,000) from Stressmarq; HSC70 (SPA-815; RRID:AB_10617277; 1:500), HOP (SRA-1500; RRID:AB_10618972; 1:1,000) from Enzo; HSP90α (ab2928; RRID:AB_303423; 1:6,000) and HSP70 (ab94368; RRID:AB_10716913; 1:5,000) from Abcam; CDC37 (4793; RRID:AB_10695539; 1:1,000) and HOP (5670; RRID:AB_10828378; 1:500), from Cell Signaling Technology.

Validation

All antibodies are commercially available and have been validated by the manufacturer. Supporting publications are found on the manufacturer's site. Relevant positive and negative controls were used to further validate several antibodies as indicated in the relevant figures. Antibodies have been validated either from prior reports and studies or validated by the manufacturer as stated on the website from the catalog numbers listed above or published references on the manufacturers' websites. Manufacturer states the antibody has been validated for intended use. Manufacturer citation are listed in manufacturer website for each specific antibody.

Eukaryotic cell lines

Policy information about cell lines

Cell line source(s)

The human breast cancer, MDA-MB-468 (HTB-132; RRID:CVCL_0419) and pancreatic cancer cell lines; ASPC-1 (CRL-1682; RRID:CVCL_0512), PL45 (CRL-2558; RRID:CVCL_3567), MiaPaCa2 (CRL-1420; RRID:CVCL_0428), SU.86.86 (CRL-1837; RRID:CVCL_3881), CFPAC (CRL-1918; RRID:CVCL_1119), Capan-2 (HTB-80; RRID:CVCL_0026), BxPc-3 (CRL-1687; RRID:CVCL_0186), HPAF-II (CRL-1997; RRID:CVCL_0313), Capan-1 (HTB-79; RRID:CVCL_0237), Panc-1 (CRL-1469; RRID:CVCL_0480) and Panc 05.04 (CRL-2557; RRID:CVCL_1637) were purchased from the American Type Culture Collection (ATCC; Manassas, VA, USA). The patient derived cells 931102 and 931019 were provided by Dr. Yelena Janjigian (MSKCC) and MSK-HR-Panc1 was provided by Dr. Vinagolu K. Rajasekhar (MSKCC). The human leukaemia cell line, HL-60 cell line (CCL-240; RRID: CVCL_0002), was purchased from ATCC

Authentication

Cell were authenticated using short tandem repeat profiling

Mycoplasma contamination

Cells were routinely tested for mycoplasma and were found to be negative

Commonly misidentified lines
(See ICLAC register)

none was used

Animals and other organisms

Policy information about studies involving animals; ARRIVE guidelines recommended for reporting animal research

Laboratory animals	All animal studies were conducted in compliance with MSKCC's guidelines and under Institutional Animal Care and Use Committee (IACUC) approved protocols #05-11-024 and #04-03-009. Female athymic nu/nu mice (Hsd:Athymic Nude-Foxn1nu, female, 20-25 g, 6 weeks old; RRID: MGI:5652489) were obtained from Envigo and allowed to acclimatize at the MSKCC vivarium for 1 week before implanting tumours. Mice were provided with food and water ad libitum. All mice in all studies were observed for clinical signs at least once daily. Mice were housed in groups of 4-5 mice per individually ventilated cage in a 12h light/dark cycle (6am/6pm), with controlled room temperature (22 ± 1oC) and humidity (30-70%). Mice were provided with food and water ad libitum. All mice in all studies were observed for clinical signs at least once daily.
Wild animals	The study did not involve wild animals
Field-collected samples	none
Ethics oversight	All procedures were approved by the MSKCC Institutional Animal Care and Use Committee.

Note that full information on the approval of the study protocol must also be provided in the manuscript.

Flow Cytometry

Plots

Confirm that:

- [x] The axis labels state the marker and fluorochrome used (e.g. CD4-FITC).
- [x] The axis scales are clearly visible. Include numbers along axes only for bottom left plot of group (a 'group' is an analysis of identical markers).
- [x] All plots are contour plots with outliers or pseudocolor plots.
- [x] A numerical value for number of cells or percentage (with statistics) is provided.

Methodology

Sample preparation	Cells in culture. Epichaperome positivity was determined using a FITC-conjugated PU-H71
Instrument	LSRFortessa (BD Biosciences)
Software	FowJo
Cell population abundance	homogeneous cell population
Gating strategy	see Supplementary fig.1

- [x] Tick this box to confirm that a figure exemplifying the gating strategy is provided in the Supplementary Information.
